# Emotional Reactions of Professionals to Challenging Behaviors in People with Intellectual and Developmental Disability

**DOI:** 10.3390/bs14080707

**Published:** 2024-08-13

**Authors:** Juliana Reyes-Martín, David Simó-Pinatella, Ana Andrés

**Affiliations:** 1 Facultat de Psicologia, Ciències de l’Educació i de l’Esport, Blanquerna, Ramon Llull University, 08022 Barcelona, Spain; davidsp@blanquerna.url.edu (D.S.-P.); anaav2@blanquerna.url.edu (A.A.); 2 Fundació Vallparadís, Mutua Terrassa, 08221 Barcelona, Spain

**Keywords:** intellectual and developmental disability, challenging behavior, professionals, emotional reactions

## Abstract

Professionals working with people with intellectual and developmental disability (IDD) can be exposed to challenging behaviors (CBs), which may result in professionals exhibiting emotional reactions that can impact their practices. This study examined these reactions and analyzed how they are influenced by the individual characteristics of people with IDD (gender, age, and level of IDD) and the variables related to CB (type of behavior and its frequency and behavioral function). A total of 125 professionals assessed 293 people with IDD who exhibited CBs. The professionals were asked to complete the Behavior Problems Inventory—Short Form, the Emotional Reactions to Challenging Behavior Scale, and the Questions About Behavior Function measure. It was revealed that positive emotional reactions predominated over negative ones. Significant results were found regarding the level of disability and the age of people with IDD. Emotional reactions were related to the severity of CBs, especially self-injurious and aggressive/destructive behavior, as well as certain behavioral functions. The severity of CBs and the age of people with IDD emerge as key predictors of the emotional reactions of professionals. In short, these reactions vary according to different variables, highlighting the importance of interventions that address professionals’ emotional needs.

## 1. Introduction

Intellectual and developmental disability (IDD) is characterized by significant limitations in intellectual functioning and adaptive skills in conceptual, social, and practical aspects that originate during the developmental period [1]. People with IDD often exhibit challenging behavior (CB) [2,3,4,5]. It is estimated that up to 60.4% of people with IDD exhibit some type of CB [6], although the prevalence of such behaviors may vary depending on the population studied, research approach, and definition of CB used [7,8,9,10]. Some of the most common CBs include self-injurious, aggressive/destructive, or stereotyped behaviors. In particular, self-injurious behavior includes actions directed toward oneself that cause damage to one’s own body, such as hitting one’s head or body. On the other hand, aggressive or destructive behavior manifests itself in deliberate acts against other people or property, such as pinching others’ or destroying objects. Likewise, stereotyped behavior is evidenced by voluntary acts that occur repeatedly in the same way, such as sniffing objects or one’s own body, or yelling and screaming [11]. For example, some studies indicate a prevalence exceeding 30% for self-injurious [12,13], over 30% for aggressive/destructive [13], and above 55% for stereotyped [14,15] behavior. These behaviors significantly impact the people who exhibit them [16,17,18], putting their safety at risk and compromising their quality of life, participation [19,20,21], and close environment [22,23,24,25].

Findings from the literature showed how the characteristics of the environment and services can influence the occurrence and types of CB in people with IDD [26,27]. The professionals who work in these services play a crucial role in intervening in and preventing such behaviors [27]. However, the occurrence of CB can result in high levels of stress and occupational burnout in professionals [23,28], which, in turn, can contribute to high turnover rates and absenteeism [29,30,31,32] and can trigger emotional reactions in professionals such as fear, anger, anxiety, and annoyance [33,34,35,36].

Because of the difficulties in measuring the emotional reactions of professionals to CB, this research has been limited in the past [37]. Mitchell and Hastings [38] developed the Emotional Reactions to Challenging Behaviors Scale (ERCBS), which included only negative emotional reactions to CB [38]. Subsequently, items corresponding to positive emotional reactions were added [39], including cheerful/excited (for example, feeling happy or invigorated) and confident/relaxed (for example, experiencing confidence or comfort). The negative emotional reactions measured included depression/anger (for example, feeling guilty or hopeless) and fear/anxiety (for example, being shocked or afraid). Subsequent studies have translated and validated this instrument in Korean [40], Dutch [41], and Spanish [36].

From the literature, it is clear that the emotional reactions of care professionals can act as a mediating factor between CB and the stress perceived by professionals [42], but they may not contribute to the prevention of this type of behavior (e.g., ignoring or avoiding the behavior [39]) or alter the interaction between people with IDD and professionals [43]. That is, the emotional reactions of professionals to CB may have a negative impact on the quality of care provided to people with IDD [44,45,46]. Willems et al. [47] revealed that professionals’ negative emotions, such as anxiety and anger, were associated with more controlling and hostile behavior, as well as greater support seeking, while positive emotions were linked with the friendlier behavior of professionals toward people with IDD.

Several factors that may influence the emotional reactions of professionals to CBs have been identified. First, the typology, frequency, and severity of the CB can affect professionals’ emotional reactions [48]. For instance, professionals tend to respond with irritation, anger, or fear to aggression and with sadness, despair, anger, irritation, and disgust to self-injury [33]. Moreover, the severity of self-injurious and aggressive/destructive behavior and the frequency of aggressive/destructive behavior have been associated with fear/anxiety in professionals. In contrast, stereotypic behavior may not always be perceived as problematic and therefore may not trigger emotional reactions in professionals [49]. Second, the function of the CB plays a significant role. [50]. As reported, self-injurious behaviors maintained through negative reinforcement are associated with more negative emotions among professionals. Third, causal attributions about behavior can influence professionals’ emotional reactions [51,52]. Dagnan et al.‘s study [52] found that professionals’ negative emotions were linked to the perceived ability to change the behavior of people with IDD, which affected the willingness of professionals to provide assistance. For example, it was observed that when professionals believed they could control behavior, they experienced fewer negative emotions [52,53]. Likewise, a study identified a trend of experiencing more negative emotions when the responsibility for the CBs was attributed to people with IDD [51]. Fourth, it has been observed that professionals with better stress management and adaptation skills experience fewer negative emotions in response to CBs. However, it is suggested that emotional intelligence seems to be more closely associated with feelings than with emotions in this specific context [41]; emotions are more instinctive and basic, while feelings are more linked to cognitive and reflective processes [54,55,56]. Lastly, the perception of the self-efficacy of professionals in managing CBs predicts the emotional reactions of both fear/anxiety and depression/anger [57], that is, professionals who have greater confidence in their ability to manage CBs tend to experience fewer negative emotional reactions. However, the exploration of how certain characteristics of people with IDD affect the emotional responses of professionals has been more limited. To the best of our knowledge, just one study suggests that age also influences this, with fewer emotional reactions manifested in services attending to adults than in those attending to children [36].

Considering the role that emotional reactions may play in the link between CB exposure and staff work stress [42], as well as their ability to affect the origin and persistence of CB [39,49], the need for greater understanding in this area is evident. It is crucial to explore the emotional reactions of professionals toward CB, considering the diversity of types and functions of these behaviors, as well as other factors. Although progress has been made, there is still limited knowledge about staff emotional reactions to CB, as well as the factors that may anticipate these reactions. It is possible that other factors, such as the individual characteristics of people with IDD, also play an important role in this regard, and identifying these factors is essential to the development of effective professional training and support programs [38,57]. These programs should integrate emotional regulation strategies into team training and supervision [47,58], not only to reduce CB and negative emotional reactions but also to mitigate stress and prevent emotional exhaustion in professionals.

The main objectives of this study are (a) to examine the emotional reactions experienced by professionals who work with people with IDD who present CB, (b) to analyze how the various characteristics of people with IDD (age, gender, and level of ID) are related to the emotional reactions of professionals, and (c) to explore how variables related to CB (types, frequency, and functions of behaviors) are related to the emotional reactions of professionals. Furthermore, this study intends to carry out a predictive analysis of the emotional reactions of professionals based on these variables related to people with IDD and CB.

## 2. Methods

### 2.1. Setting and Participants

A total of 125 professionals (88% women) from Spanish care services for people with IDD participated in this study, ranging in age from 20 to 62 years (M = 43.66, SD = 11.64). The sample was diversified across different work settings, including special education schools (49.6%), residential care homes or daycare centers (40.8%), occupational therapy centers (8.8%), and leisure and recreation services (0.8%). Regarding their educational level, 52% had a bachelor’s degree, 32% had completed secondary education (high school or equivalent), 15.2% had a master’s degree or postgraduate studies, and 0.8% had completed primary education. Additionally, 81% had specific training in addressing CB, such as positive behavioral support. In terms of professional roles, 47.2% were tutors or schoolteachers, 37.6% worked as educators or direct support staff, 8.8% were psychologists, 3.2% were service directors, 1.6% were educational psychologists, 0.8% were speech and language therapists, and 0.8% were engaged in other areas. Regarding their work in the field of IDD, they had an average of 15.12 years (SD 11) of experience, with an average of 11.4 years (SD 10.08) in their current position. To participate in this research, professionals were required to (a) have a minimum of one year of experience in the field of IDD and CB and (b) have known the person being evaluated for at least six months. Professionals not meeting these requirements were excluded. Also, professionals who did not have a direct care role managing CBs in people with IDD were excluded. On average, they had known the person being evaluated for five years and nine months and had contact five days a week.

In addition to the professionals, a total of 293 people with IDD (33.45% women) were assessed by these professionals. Some professionals tested more than one person with IDD. To be included in the study, people with IDD had to meet certain requirements: (a) have a formal diagnosis of IDD, (b) be over 6 years old, and (c) have exhibited CB (aggressive/destructive, self-injurious, or stereotyped behaviors) in the last two months. People with IDD not meeting these criteria were excluded. People with IDD ranged in age from 6 to 74 years (M = 31.89, SD = 17.22). Regarding the level of IDD, 6.14% were classified as mild, 24.23% as moderate, 50.17% as severe, and 19.45% as profound. Additionally, most participants had other diagnoses, such as autism spectrum disorder. Regarding CB, 71.33% exhibited self-injurious behaviors, 85.67% exhibited aggressive/destructive behaviors, and 87.71% exhibited stereotyped behaviors. Table 1 provides additional information on the typologies of CBs exhibited by people with IDD and their sociodemographic information.

### 2.2. Outcome Measures

Participating professionals completed the following battery of questionnaires:

Demographic data of people with IDD: This questionnaire has questions regarding the individual being evaluated, specifically information about their age, gender, level of IDD, degree of IDD, level of adaptive behavior, associated diagnoses, services attended, and place of residence.

The Behavior Problems Inventory—Short Form (BPI-S) [11,59]: This evaluates the frequency and severity of CBs observed in people with IDD during the last two months. It consists of three subscales: self-injurious behavior, aggressive/destructive behavior, and stereotypical behavior. It is scored using two Likert-type scales: one for frequency, ranging from 0 (“never”) to 4 (“every hour”), and another for severity, ranging from 0 (“no problem”) to 3 (“serious problem”). The score ranges for each of the BPI-S subscales are as follows: self-injurious behavior (frequency: 0–28; severity: 0–21); aggressive/destructive behavior (frequency: 0–36; severity: 0–27); and stereotyped behavior (frequency: 0–48). For the present study, the validated Spanish version was used.

The Emotional Reactions to Challenging Behavior Scale (ERCBS) [38,39]: This instrument evaluates the emotional reactions of professionals to CB in people with IDD. For this study, the Spanish version of the ERCBS [36] was used. As with the original instrument, it is composed of 23 items. However, in the Spanish version, they are grouped around two factors: one related to negative emotional reactions and another to positive emotional reactions. Professionals rate their emotional experience on a Likert-type scale ranging from 0 (“no, never”) to 3 (“yes, very often”). The minimum and maximum scores for each subscale are as follows: negative emotional reactions (0–45) and positive emotional reactions (0–24).

Questions About Behavior Function (QABF) [60]: This instrument aims to determine the function of CB in people with IDD. The questionnaire, conducted in an interview format, consists of 25 questions grouped into 5 factors (attention, escape, sensory, physical, and tangible). Participating professionals are required to rate the likelihood that CB is related to each factor on a Likert-type scale from 0 to 3, with 0 indicating “never” and 3 “often”. Each subscale is scored for both frequency and severity, with frequency ranging from 0 to 5 and severity ranging from 0 to 15. The Spanish-translated and validated version was used.

### 2.3. Procedure

The project was approved by the Faculty’s Ethics and Research Committee (Ref. 2122012D). In accordance with the Code of Ethics, the confidentiality of each participant’s data was maintained. Written informed consent was obtained from participating professionals as well as from the people with intellectual disabilities on whom they reported and from the guardians or family of those people. This ensured that all ethical considerations related to this research were addressed and that only those who consented participated in this study.

Regarding the recruitment procedure, the research team contacted the centers to invite their participation in this study. They provided detailed information about the research objectives and structure and encouraged the centers to take part. Upon agreement to participate, the centers were asked to identify people with IDD who met the inclusion criteria, and their consent was obtained. After obtaining consent from all participants, professionals completed a battery of instruments digitally using SurveyMonkey software (SurveyMonkey is copyrighted from 1999 to 2024. The survey was created and first accessed on 20 June 2022, and remained active until 10 July 2023. The platform URL link is https://www.surveymonkey.com). The administration of the test battery followed a predetermined order: first, participants completed the BPI-S to assess the frequency and severity of CB observed in the last two months. Subsequently, they completed the ERCBS [36], rating their own emotional reactions to the CB of the person with IDD. Finally, they completed the QABF to assess the function of the behavior. If people with IDD presented more than one CB, the one with the highest frequency score on the BPI-S [11] was evaluated. Participants received detailed instructions on how to complete each instrument and were guided on the interpretation of the questions and response scales. Once all data were collected through the digital platform, statistical analysis was conducted.

### 2.4. Statistical Analyses

Data were analyzed using IBM SPSS Statistics version 24 (IBM Corp., Armonk, NY, USA) and IBM SPSS Amos Statistics version 20 (IBM Corp., Chicago, IL, USA).

A descriptive analysis of the variables was conducted in terms of central tendency (means), dispersion (standard deviations), and distribution (percentages), specifically, the negative and positive emotional reactions of professionals to the CBs of people with IDD examined through the ERCB and the frequency and severity of self-injurious, aggressive/destructive behavior and frequency of stereotypic behavior assessed using the BPI-S.

A Wilcoxon T test was applied to compare scores obtained on the ERCB and examine specific differences between the scores of positive and negative emotions. To analyze the differences in the means of emotional responses based on various variables, non-parametric Mann–Whitney U and Kruskal–Wallis tests were conducted to assess their relationship with gender and IDD level, respectively. The analysis of the relationship between various quantitative variables was carried out using Spearman correlations. The effect size interpretation of correlation coefficients followed Cohen’s [61] criteria, with correlations between 0.1 and 0.3 classified as small, those between 0.3 and 0.5 as medium, and those between 0.5 and 1.0 as large.

A multiple regression analysis was performed in hierarchical thematic blocks to evaluate the predictors of negative and positive emotional reactions. For this analysis, participants with outliers were excluded. Those participants whose residuals were 3 or more standard deviations from the mean were considered outliers. Data independence was verified using the Durbin–Watson statistic, and multicollinearity was assessed using tolerance statistics and the variance inflation factor, confirming that there was no multicollinearity in the data. Assumptions of homoscedasticity, normality in the error distribution, and the absence of multicollinearity were tested using Durbin–Watson statistics, tolerance statistics, and the variance inflation factor.

## 3. Results

### 3.1. Emotional Reactions of Professionals

Table 2 presents how professionals feel when people with IDD engage in CBs, including the means and standard deviations of the scores obtained in the subscales of negative emotional reactions, positive emotional reactions, and ERCBS total scores. Additionally, it includes the means, standard derivations, and percentages of negative and positive emotional reactions (items of the scale) experienced by each professional to the CB presented by people with IDD.

Positive emotions obtained a significantly higher mean score than negative emotions (W = 13693, *p* < 0.001). For negative reactions, descriptive analysis revealed a median of 5, and the interquartile range for negative reactions was 7, with a first quartile of 3 and a third quartile of 10. Regarding positive reactions, the median was 8, and the interquartile range was 8, with a first quartile of 4 and a third quartile of 12. The items that obtained the highest mean scores were “self-assured” and “confident”, while those with the lowest were “excited” and “happy”.

Regarding negative emotions, the items that obtained the highest mean scores were “disgusted” and “nervous”, whereas those with the lowest were “humiliated” and “betrayed” on these subscales.

### 3.2. Emotional Reactions of Professionals Related to Age, Gender, and Level of IDD

As shown in Table 3, statistically significant negative correlations were identified between the age of people with IDD and the negative emotional reactions of professionals. Significant positive correlations were also observed between the age of people with IDD and positive emotional reactions.

No significant differences between the emotional reactions of professionals toward the gender of the participants exhibiting CB were observed (see Table 4). Only significant effects of the gender of people with IDD on the ERCB total score were identified.

In relation to the level of IDD, no significant effects of the different levels of IDD (mild, moderate, severe, or profound) on the negative emotional reactions of professionals were found. However, significant differences were identified in the positive emotional reactions of professionals according to the levels of IDD (see Table 5). Specifically, discrepancies were observed between the moderate and severe levels, as well as between the moderate and profound levels in positive reaction scores.

### 3.3. Emotional Reactions of Professionals Related to Problem Behavior

Table 3 displays the correlations among the scores of the negative and positive emotional reaction subscales, the total ERCB score, and several variables related to the type and function of CB.

The negative emotional reaction subscale score exhibited significant and positive correlations with the frequency of self-injurious behavior, while no significant correlation was found with its severity. Additionally, it showed significant positive correlations with both the frequency and severity of aggressive and destructive behavior. Conversely, negative emotional reactions did not correlate with the frequency of stereotyped behavior.

No significant correlations were found between positive emotional reaction scores and the frequency and severity of self-injurious behavior. However, significant negative associations were observed between positive emotional reaction scores and both the frequency and severity of aggressive and destructive behavior. Positive emotional reactions did not correlate with the frequency of stereotyped behavior.

The ERCB total score displayed positive correlations with the frequency of self-injurious and stereotyped behaviors.

Regarding behavioral function, the results revealed significant associations between the total negative emotional reactions score and various behavioral functions, including attention-seeking, escape, and obtaining tangible objects. Nonetheless, no correlations were observed with sensory function or physical pain. Notably, the positive emotional reaction score did not demonstrate a significant correlation with any behavioral function in this study. Finally, the ERCB total score positively correlated with attention-seeking, escape, and obtaining tangible objects.

### 3.4. Predictors of the Negative and Positive Emotional Reactions of Staff

Two hierarchical regression analyses were conducted to examine negative and positive emotional reactions, incorporating three blocks of variables: type of CB, function of CB, and characteristics of people with IDD.

In the case of negative emotional reactions, the final model accounted for 18% of the variability in these reactions. Significant variables included the severity of aggressive/destructive behaviors, frequency of self-injurious behaviors, escape, and attention functions, and the age of people with IDD. An F-test confirmed the statistical significance of this model (see Table 6).

In relation to positive emotional reactions, the final model successfully predicted 15% of the variability in these responses. Significant variables included those related to the severity and frequency of aggressive and destructive behaviors, as well as the age of people with IDD. Additionally, the F-test confirmed the statistical significance of the final model, which incorporated these pertinent variables (see Table 7).

## 4. Discussion

The present study explored professionals’ emotional reactions to CBs presented by people with IDD. Furthermore, we sought to identify the variables associated with these emotional reactions, as well as potential predictors of the positive and negative emotional responses that professionals exhibit toward CBs.

The findings of this study show a trend toward positive emotional responses to CBs by professionals. However, it is essential to recognize the presence of reported negative emotional reactions, as they may indicate areas where professionals may need further support or training to effectively manage these difficult situations. In this way, the positive reactions with the highest average scores were “self-assured”, “confident”, and “comfortable”, while the negative ones were “disgusted”, “nervous”, and “frustrated”. In contrast, previous studies had identified mostly negative emotions, such as fear, anger, and frustration, and to a lesser extent, positive emotional reactions [33,34,50,51,62]. This discrepancy could be attributed to variations in the assessment instruments used. For example, it was not until 2003 that the ERCB [38] was expanded to include positive emotional responses [39], allowing for a more complete and balanced assessment [63,64]. Furthermore, the literature notes that variables such as the support and resources available (i.e., additional professionals, specialized training, and skills of the professionals) [38], as well as the quality of the relationship between the professional and the people with IDD, can influence the reduction in negative emotional reactions. A strong, positive relationship can mitigate the impact of CBs, while a tense or conflictive relationship can intensify negative emotions [38].

When examining the personal characteristics of people with IDD and their influence on the emotional reactions of professionals to CBs, we found no significant role played by the gender of people with IDD. Conversely, both age and the level of IDD significantly impacted the emotional reactions of professionals. Specifically, professionals tend to experience fewer negative emotional reactions as individuals with intellectual disabilities age. Furthermore, the results show that, regardless of the level of IDD, professionals experience similar levels of negative emotional reactions. However, when analyzing the positive emotional reactions of professionals, significant differences were found according to the levels of IDD, suggesting that the specific characteristics of each level of IDD can influence how professionals perceive and experience positive emotions in their work. In general, professionals who worked with people with severe and profound IDD tended to show higher levels of positive affectivity compared to those who worked with people with a moderate level of IDD. Montañés et al. [36] highlight that professionals who work with adults show lower levels of depression, anger, fear, and anxiety compared to those who work in early care services for younger people. Furthermore, professionals show more negative responses to the problematic behaviors presented by people with mild and moderate IDD compared to those with profound IDD [47]. In our sample, only statistically significant differences were detected between gender and the severity of self-injurious behavior, with the mean of the women/girls group being higher than that of the men/boys group. This could be due, in addition to the equitable training of professionals, to an egalitarian perception of behaviors and the context of care focused on gender equity. On the other hand, and in relation to the level of IDD, professionals who work with people with more severe IDD could develop deeper and more meaningful relationships with the people they care for due to the more intense and prolonged nature of their interaction. These relationships can generate a greater satisfaction and positive affect at work.

Furthermore, concerning variables associated with CBs (type, frequency, and function), a correlation with emotional responses was observed. In line with the existing literature, our results coincide with previous studies that have found a relationship between the negative emotional reactions of professionals and the frequency of self-harming behaviors [34] and the frequency and severity of aggressive/destructive behaviors [34,43]. However, a similar correlation was not observed with stereotypic behaviors [34,43,65]. In contrast to the findings of Lambrechts et al. [43], who reported a correlation between negative emotional reactions and the severity of self-harming behaviors, our study did not detect a significant correlation in this aspect.

Similarly, positive emotional responses were not directly related to self-injurious or stereotyped behaviors. However, we found negative correlations between negative emotional reactions and the frequency or severity of aggressive/destructive behaviors. Professionals seem to find externalizing behaviors, such as aggressive/destructive, more challenging compared to self-directed behaviors, such as self-injurious or stereotyped [66]. Furthermore, professionals perceive stereotypical behaviors as less annoying, probably because they do not represent a direct threat or generate significant discomfort in the work environment [34,43]. In terms of behavioral function, negative emotional reactions were more present when CBs aimed to obtain tangible objects, avoid situations, or attract attention. However, no significant relationships were found between negative emotional responses and CBs that had the purpose of seeking sensory stimulation or physical pain. According to Mossman et al. [50], those behaviors that have a more social function, such as the escape function, tend to provoke intense negative emotional reactions. Furthermore, it is interesting to note that stereotyped behaviors do not seem to be perceived as problematic [43]. Based on the results, this type of behavior does not elicit positive or negative emotional reactions from the professionals caring for them. Undoubtedly, the knowledge about CBs and their purpose [50], along with the training and experience professionals have in managing behaviors, can influence these results [67,68].

Finally, and based on the discussed results, various factors can predict the emotional reactions of professionals working with people with IDD. Specifically, a greater severity in aggressive/destructive behaviors, a higher frequency of self-injurious behaviors, and attention- and escape-based behaviors are associated with a higher likelihood of professionals experiencing negative emotional reactions, such as stress, anxiety, or frustration. On the other hand, as the severity of aggressive/destructive behaviors decreases and the frequency increases, staff are more likely to experience positive emotional reactions. Similarly, as people with IDD age, negative emotional reactions seem to decrease and positive reactions seem to increase among professionals. Undoubtedly, the emotional responses of professionals to CBs can vary, forming a contextual relationship influenced by their typology, severity, and function [34,69].

This research highlights the need for interventions that address both the emotional needs of professionals as well as the CBs [70]. It is essential to consider the emotional reactions of professionals in the design of training and intervention programs. Such programs should allow preventive action (negative emotional reactions can become an early indicator of mental health problems and burnout) [70] and should enable a more effective and personalized approach [38,50]. More research is needed in this area. Future research could include how the professional setting or organization of the participants and the organizational support available can influence emotional reactions. It is essential to recognize and address the emotional needs of professionals, especially considering that stress comes more from a lack of organizational support than from direct interaction with people with IDD and CBs [71,72,73]. In this sense, it is necessary to invest in practices and research that focus on interventions aimed at breaking the cycle of interaction between CBs and their associated emotional reactions [74] and that include emotional regulation techniques in the training and supervision of professional care [47]. The continuous development of knowledge and skills over time contributes to a better capacity for management and adaptation in addressing CBs [67].

Dealing with CBs is not easy, and professionals may experience primary emotions, such as fear, which are subsequently transformed into more reflective, personal, and lasting feelings, such as helplessness [33,38,41]. Adopting the approach outlined in this paper in future research will provide the scientific community and direct care professionals with a better understanding of how the primary emotions experienced by staff are converted into more personal feelings.

### Limitations

The results of this study should be understood considering two main limitations. First, the emotional reactions of professionals to problem behaviors were assessed using the ERCBS, a self-report measure. In this sense, evaluations based on self-reports may be influenced by social desirability, where subjects tend to provide answers that they consider socially acceptable rather than truthful answers [75]. Second, in this study, as in the literature in general, emotions and feelings were addressed in a similar way [38]. As previously explained, emotions are more instinctive and basic, while feelings are more linked to cognitive and reflective processes [54,55,56].

## 5. Conclusions

The emotional reactions of professionals to CBs can vary significantly depending on the specific nature of these behaviors, which establishes a highly contextual relationship influenced by the type, severity, and function of the behavior, as well as by certain characteristics of the people with IDD. These findings underscore the importance of implementing interventions that address both the emotional demands of professionals and the needs of people with IDD, emphasizing ongoing training and emotional management.

## Figures and Tables

**Table 1:** Typologies of challenging behaviors (CBs) and sociodemographic information of individuals with intellectual and developmental disability (IDD) reported by participating professionals (n = 293).

	Prevalence N (%)	Frequency Mean (SD ^4^)	Severity Mean (SD)
CBs exhibited by individuals with IDD			
SIB ^1^	209 (71.33)	3.47 (3.5)	2.3 (2.37)
ADB ^2^	251 (85.67)	5.73 (4.94)	4.76 (4.2)
SB ^3^	257 (87.71)	13.4 (10.07)	-
Only SIB	6 (2.05)	-	-
Only ADB	17 (5.8)	-	-
Only SB	9 (3.07)	-	-
SIB and ADB	12 (4.1)	-	-
SIB and SB	25 (9.56)	-	-
ADB and SB	55 (18.78)	-	-
Other diagnoses of individuals with IDD			
Autism spectrum disorder	120 (40.96)	-	-
Seizure disorder	34 (11.6)	-	-
Sensory impairment (auditory and/or visual)	35 (11.95)	-	-
Physical disability	47 (16.04)	-	-
Attention deficit hyperactivity disorder	12 (4.1)	-	-
Down syndrome	11 (3.75)	-	-
Other genetic syndrome	51 (17.41)	-	-
Mental health disorder	29 (9.9)	-	-
Brain damage	16 (5.46)	-	-
Cerebral palsy	26 (8.87)	-	-
Other diagnosis or unknown	20 (6.83)	-	-
Level of adaptive behavior of individuals with IDD			
Intermittent support	4 (1.37)	-	-
Limited support	3 (1.02)	-	-
Intermittent or limited support + conduct disorder	25 (8.53)	-	-
Extensive support	31 (10.58)	-	-
Generalized support	67 (22.87)	-	-
Generalized Support + conduct disorder	114 (38.91)	-	-
Unknown	49 (16.72)	-	-
Residential placement			
Home family	125 (42.66)	-	-
Residential care home	158 (53.93)	-	-
Residence or group home	7 (2.39)	-	-
Others	3 (1.02)	-	-
Specialized care service			
Special education school	106 (36.18)	-	-
Occupational therapy centers	25 (8.53)	-	-
Day care centers	27 (9.22)	-	-
Residential care home	131 (44.71)	-	-
Others	4 (1.37)	-	-

Note: ^1^ self-injurious behavior; ^2^ aggressive or destructive behavior; ^3^ stereotyped behavior; ^4^ standard deviation. Frequency and severity of CBs assessed with the Emotional Reactions to Challenging Behavior Scale (ERCBS): 0–45 for negative emotional reactions and 0–24 for positive reactions.

**Table 2:** Descriptive statistics for the ERCBS across all 125 participating professionals.

ERCBS	Mean (SD)	Never (%)	Yes, Sometimes (%)	Yes, Frequently (%)	Yes, Very Frequently (%)
Negative affectivity subscale	6.43 (5.21)	-	-	-	-
1. Shocked	0.45 (0.73)	66.89	22.18	8.19	2.05
3. Guilty	0.22 (0.51)	81.91	14.68	2.39	0.68
4. Hopeless	0.62 (0.82)	56.31	28.33	11.6	3.41
6. Afraid	0.3 (0.61)	76.11	19.11	2.73	1.71
7. Angry	0.56 (0.76)	56.63	31.4	10.58	2.05
9. Incompetent	0.22 (0.53)	81.91	14.33	2.39	1.02
11. Frustrated	0.74 (0.84)	47.44	33.79	14.02	3.41
12. Helpless	0.27 (0.6)	80.21	13.31	5.12	1.03
14. Disgusted	0.8 (0.83)	43	35.84	17.41	3.07
16. Resigned	0.69 (0.92)	56.31	22.87	14.68	5.46
17. Frightened	0.23 (0.52)	80.89	14.33	3.75	0.34
19. Humiliated	0.05 (0.23)	95.22	4.1	0.34	0
20. Betrayed	0.06 (0.33)	96.24	2.05	0.68	0.34
21. Sad	0.45 (0.69)	65.19	25.94	7.17	1.37
23. Nervous	0.78 (0.79)	40.96	41.98	13.99	2.73
Positive affectivity subscale	8.65 (5.63)	-	-	-	-
2. Confident	1.79 (1.03)	17.75	11.26	43.35	25.94
5. Comfortable	1.09 (1.12)	44.05	13.99	26.96	13.31
8. Invigorated	0.71 (1.01)	61.43	11.26	19.11	6.83
10. Happy	0.7 (1.06)	65.53	8.53	16.04	9.56
13. Self-assured	1.97 (0.93)	10.92	12.29	45.73	30.72
15. Relaxed	1.07 (1.08)	42.32	19.45	24.92	12.29
18. Cheerful	0.93 (1.09)	53.24	8.53	28.67	8.87
22. Excited	0.48 (0.83)	70.31	13.99	12.29	3.07
Total ERCB	15.09 (6.45)				

**Table 3:** Spearman correlations between the emotional reactions of participating professionals and the characteristics (age, type, and function of CB) of individuals with IDD and CB on whom they reported.

	Negative Affectivity Subscale	Positive Affectivity Subscale	Total ERCB
Age of the participants with IDD	−0.25 ***	0.27 ***	0.04
Challenging behavior			
SIB-Frequency	0.13 *	0.05	0.15 **
SIB-Severity	0.11	0.00	0.09
ADB-Frequency	0.19 ***	−0.12 *	0.06
ADB-Severity	0.21 ***	−0.25 ***	−0.04
SB-Frequency	0.04	0.09	0.12 *
Behavioral function			
Attention	0.26 ***	−0.07	0.14 *
Escape	0.28 ***	−0.04	0.18 **
Sensorial	−0.04	0.09	0.04
Physical	0.11	−0.01	0.06
Tangible	0.3 ***	−0.11	0.13 *

Note: * *p* < 0.05; ** *p* < 0.01; *** *p* < 0.001.

**Table 4:** Comparison of the means of emotional reactions between women/girls and men/boys.

ERCB	Women/Girls (N = 98) M (SD)	Men/Boys (N = 195) M (SD)	Mann–Whitney U Test
Negative affectivity subscale	6.5 (4.9)	6.4 (5.37)	9188.5 *p* = 0.59
Positive affectivity subscale	9.66 (5.99)	8.14 (5.39)	8256.5 *p* = 0.06
Total ERCB	16.16 (6.23)	14.54 (6.5)	8120.5 *p* = 0.04

**Table 5:** Comparison of the mean scores of emotional reactions by level of IDD.

ERCB	Group I ^1^ (N = 18) Mean (SD)	Group II ^2^ (N = 71) M (SD)	Group III ^3^ (N = 147) M (SD)	Group IV ^4^ (N = 57) M (SD)	Kruskal– Wallis	Dunn’s Post Hoc Test Comparisons-Level IDD
Negative affectivity subscale	7.67 (3.76)	7.62 (5.94)	5.89 (5.04)	5.97 (4.85)	(3, 7.51) *p* = 0.06	-
Positive affectivity subscale	6.67 (3.34)	6.78 (5.43)	9.44 (5.63)	9.6 (5.87)	(3, 14.84) *p* = 0.002	II < III *** II < IV **
Total ERCB	14.33 (3.94)	14.39 (7.14)	15.33 (6.39)	15.56 (6.38)	(3, 1.26) *p* = 0.74	-

Note: ^1^ mild level; ^2^ moderate level; ^3^ severe level; ^4^ profound level; ** *p* < 0.01; *** *p* < 0.001.

**Table 6:** Multiple linear regression model using thematic blocks to predict negative affectivity.

	Negative Affectivity Subscale
Block 1	
ADB-Severity β ^1^	0.22 *
ADB-Frequency β	0.03
SIB-Frequency β	0.18 **
R^2^	0.1
Adjusted R^2^	0.09
F	(3.287) 11.09 ***
Block 2	
ADB-Severity β	0.14 *
SIB-Frequency β	0.18 **
Tangible β	0.07
Escape β	0.15 *
Attention β	0.13 *
R^2 2^	0.17
Adjusted R^2 3^	0.15
Δ Adjusted R^2 4^	0.06
F ^5^	(5.285) 11.51 ***
Block 3: final model	
ADB-Severity β	0.13 *
SIB-Frequency β	0.18 **
Escape β	0.15 *
Attention β	0.15 **
Age of person with IDD	−0.15 **
R^2^	0.18
Adjusted R^2^	0.17
Δ Adjusted R^2^	0.02
F	(5.285) 12.9 ***

Note: ^1^ beta regression coefficient; ^2^ coefficient of determination; ^3^ adjusted R-squared; ^4^ change in adjusted R; ^5^ F-statistic; * *p* < 0.05; ** *p* < 0.01; *** *p* < 0.001.

**Table 7:** Multiple linear regression model using thematic blocks to predict positive affectivity.

	Positive Affectivity Subscale
Block 1	
ADB-Severity β	−0.48 ***
ADB-Frequency β	0.28 **
R^2^	0.1
Adjusted R^2^	0.09
F	(2.288) 15.33 ***
Block 2	
ADB-Severity β	−0.41 ***
ADB-Frequency β	0.26 **
Age of person with IDD β	0.21 ***
Level IDD β	0.07
R^2^	0.15
Adjusted R^2^	0.14
Δ Adjusted R^2^	0.05
F	(4.286) 12.83 ***
Block 3: final model	
ADB-Severity β	−0.41 ***
ADB-Frequency β	0.26 **
Age of person with IDD β	0.21 ***
R^2^	0.15
Adjusted R^2^	0.14
F	(4.286) 16.64 ***
	Positive affectivity subscale

Note: ** *p* < 0.01; *** *p* < 0.001.

## Data Availability

Data supporting the findings of this study can be obtained from the corresponding author upon reasonable request.
